# Non-union of the ulnar styloid process in children is common but long-term morbidity is rare: a population-based study with mean 11 years (9–15) follow-up

**DOI:** 10.1080/17453674.2019.1596561

**Published:** 2019-04-04

**Authors:** Linda Korhonen, Sarita Victorzon, Willy Serlo, Juha-Jaakko Sinikumpu

**Affiliations:** a Department of Children and Adolescents, Pediatric Surgery and Orthopedics, Oulu University Hospital, Oulu;;; b Medical Research Centre Oulu; PEDEGO Research Group; Oulu Childhood Fracture and Sports Injury Study; University of Oulu, Oulu;;; c Department of Radiology, Vaasa Central Hospital, Vaasa, Finland

## Abstract

Background and purpose — Fracture of the ulnar styloid process (USP) is common in children in connection with distal radius fracture. The long-term morbidity of USP non-union following a childhood distal radius fracture is unclear. We evaluated long-term clinical and radiographic findings of USP non-union.

Patients and methods — All 208 children (< 16 years) who had suffered from distal radius fracture with or without a diagnosed concomitant ulnar fracture during 1992–1999 in the study institution were invited to follow-up at mean of 11 years (9–15) after the injury. Radiographs of both wrists of all 139 participants (67%) were taken; 22 patients showed USP non-union and they made up the study population. Distal radioulnar joint (DRUJ) instability, decreased range of motion (ROM), and weakened grip strength as compared with the uninjured side were the main functional outcomes. Elements of the “Disability of Arm, Shoulder and Hand” questionnaire were used for subjective symptoms.

Results — The rate of USP non-union following childhood distal forearm fracture was 16% (22/139) and only 9 of the ulnar styloid fractures were visible in the radiographs primarily. At follow-up wrist flexion–extension ROM and ulnar and radial deviation ranges did not differ between the injured and uninjured sides. Grip strengths were similar. 6 patients reported pain during exercise. 7 had ulna minus (mean 2.3 mm) but none showed degenerative radiographic findings.

Interpretation — The long-term clinical results of USP non-union following a childhood wrist fracture were good. However, one-third of the patients with USP non-union had ulnar shortening, which may predispose them to degenerative processes later in life.

The distal radius is the most common site of fracture in children and its incidence has increased during the past 40 years (Khosla et al. 2003, de Putter et al. 2011, Kazemian et al. 2011). Displacement in the fracture may be associated with interruption of the distal radioulnar ligaments and avulsion of the ulnar styloid process (USP). USP fracture is a common finding in connection with distal radius fracture in children (up to 30¬–50% of all cases) (Gogna et al. 2014, Wijffels et al. 2014). In adults, the reported rate of USP fracture in connection with distal radius fracture is even higher (Kramer et al. 2013). However, the distal ulnar epiphysis is cartilaginous and apparent not earlier than at the age of 5–9 years, with the result that USP fracture may be under-diagnosed in children (Bae and Waters 2006, Abid et al. 2008).

A USP fracture seldom justifies operative treatment (Logan and Lindau 2008, Souer et al. 2009, Chen et al. 2013, Zoetsch et al. 2013), which is mostly based on the need for surgery of the fractured radius. Casting the wrist in ulnar inclination in order to minimize USP dislocation has been suggested as a treatment option. Regardless of treatment, USP fracture frequently fails to unite (Abid et al. 2008, Gogna et al. 2014) but the risk factors of non-union are unclear.

There are several potential complications in USP non-union. The triangular fibrocartilage complex (TFCC) and anatomic bone congruity are the main factors contributing to the stability of the distal radioulnar joint (DRUJ) (Kazemian et al. 2011) and even minor changes in ulnar length can change the axial loads on the TFCC (Bae and Waters 2006). Growth arrest resulting from distal radius fracture appears as ulnar lengthening (ulna plus) (Schuurman et al. 2001, Waters et al. 2002). Respectively, ulnar shortening is a result of growth arrest of ulna and it may result in TFCC degeneration and rupture (Nelson et al. 1984). Thus, one of the most disabling complications after distal radius fracture is instability in DRUJ (Daneshvar et al. 2014, Gogna et al. 2014). In addition, chronic ulnar sided wrist pain (Yuan et al. 2017) and higher Disabilities of the Arm, Shoulder and Hand (DASH) scoring have been reported; nevertheless, they are still slight enough to fall outside clinical importance in short-term follow-up (Kazemian et al. 2011, Kramer et al. 2013, Wijffels et al. 2014, Mulders et al. 2018).

To our knowledge, most studies concerning the clinical importance of childhood USP non-union are based on short-term outcomes (Kramer et al. 2013, Gogna et al. 2014, Mulders et al. 2018) and the understanding of long-term outcomes is insufficient (Cannata et al. 2003). This study was performed to determine the rate of USP non-union and to evaluate the clinical and radiographic recovery of USP non-union in children with former distal radius fracture in long-term follow-up.

## Patients and methods

### Study design

**Figure 1.**
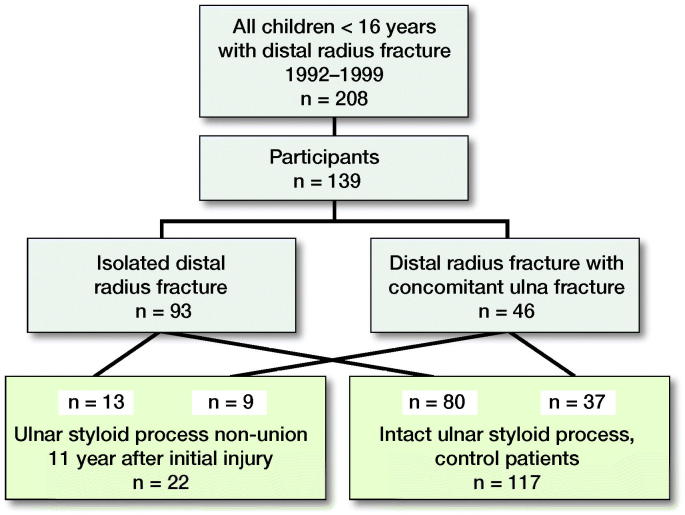
Enrollment of the patients.

This is a population-based study including all children of < 16 years of age who had suffered from distal radius fracture with or without a concomitant USP fracture and had been treated at Vaasa Central Hospital, Finland, in 1992–1999. All these patients were identified in the hospital database and they were invited to a long-term follow-up visit by letter. In cases of no-show, another letter was sent and finally the correct postal address was ensured by a nurse via a phone call. 139 patients took part out of 208 enrolled (participation 67%), at a mean of 11 years (9–15) after the injury (Figure 1). 39 out of 69 nonparticipants reported on their own initiative that they have no symptoms/no reasons to participate, while the other 30 declared no single reason (Table 1).

**Table 1.** Basic characteristics of participants (n = 139) and non-participants (n = 69)

Factor	Participants (n = 139)	Non-participants (n = 69)	p-value
Male sex, n (%)	82 (60)	48 (70)	0.2^a^
Age (SD) [range]	9.6 (3.4) [3–16]	10 (3.4) [2–16]	0.5^b^
Injury, left sided, n (%)	72 (52)	36 (52)	1.0^a^
Operation rate, n (%)	44 (32)	21 (30)	0.8^a^
Immobilization, weeks (SD) [range]	3.3 (0.8) [1–5]	3.4 (0.7) [2–4]	0.9^b^

a Standardized Normal Distribution tests.

b Independent samples t-test.

Original radiographs and hospital charts were re-reviewed to establish the baseline characteristics of the patients, injury mechanism, clinical findings, and primary treatment. The displacement and angular deformity of radius fractures were determined. The USP fractures were classified as base and tip fractures.

### Outcome variables

The rate of USP non-union was evaluated. Distal RUJ instability, decreased range of motion (ROM), and grip strength as compared with the uninjured side were analyzed as primary outcome variables. Measurements were made with a universal goniometer and the results were reported as mean values (SDs). Grip strength was measured using a hydraulic Jamar grip dynamometer (Sammons Preston, Bolingbrook, IL, USA). The best of 3 attempts was recorded. Both injured and uninjured sides were examined. To evaluate current symptoms, baseline functional scores in DASH questionnaires were used and the patients were asked about pain, tolerance in physical activity, and daily-life-related complaints. Current symptoms were compared between the patients with USP non-union and patients with absence of USP non-union. In addition, possible risk factors of USP non-union were evaluated. To evaluate the radiographic outcomes, both upper extremities were examined, except in cases of graviditas. The presence of USP non-union was recognized; its displacement (mm), and possible ulnar shortening (mm) or lengthening (mm) in the injured extremity were measured. Decreased joint space, osteophytes, and subchondral bone cysts were taken as signs of early degeneration.

### Patients

The 139 patients were on average 9.6 years (3–16) of age at the time of the injury. 60% were boys and 93% right-handed. The most usual type of injury was a fall from < 1 m (58%) and 30% fell from > 1 m. 7% resulted from traffic accidents and 4% miscellaneous injuries were found.

3% suffered from open fracture, 2 of which were Gustilo type I, and 2 of type III. The fractures were complete in 12%. Based on the primary radiographs, major (≥ 15°) angular deformity in the radius was found in 32%, slight deformity (< 15°) in 30% and no deformity in 37%. Anterior-posterior displacement of the radius was ≥ 2 mm in 26% and 0–2 mm in 6%, while coronal plane displacement (≥ 2 mm or 0–2 mm) was found in 8% and 4%, respectively. 3% of the fractures were comminuted.

USP non-union was found in 16% (22/139) of the cases, of which 9 were originally diagnosed as “distal radius fracture with concomitant ulna fracture” and 13 were isolated radius fractures; the USP fracture was invisible in the primary radiographs. According to primary and long-term radiographs, 12 of the USP fractures were at the base and 10 at the tip.

### Statistics

Statistical analysis included a chi-square test to evaluate differences between independent groups with categorical data; Fisher’s exact test was used with small groups (< 5 cases). Independent samples t-tests were used to compare differences between continuous variables. Differences of proportions were evaluated by using the binomial standardized normal deviate (SND) test. Binary logistic regression analysis was used to evaluate the potential predictive factors concerning the risk of non-union. Odds ratios (ORs) with their 95% confidence intervals (CIs) were determined in connection with age (per year of age), sex, severity of primary injury (> 2 mm displacement primarily or > 15° of angular deformity), concomitant ulnar fracture visible in radiographs, ulnar styloid fracture type (base vs. tip), open fracture (no/yes), operative treatment of radius fracture (no/yes), junior vs. senior operating surgeon and longer vs. shorter time of immobilization (≥ 28 or < 28 days) in order to identify the factors associated with USP non-union. SPSS version 24 software (IBM Corp, Armonk, NY, USA) and Stats Direct Ltd. 2013 version 3.1 (Sale, Cheshire, UK) were used. Less than 5% was considered to be the relevant level of statistical significance (p < 0.05).

### Ethics, funding, and potential conflicts of interest

The Ethics Board of Vaasa Central Hospital approved the study in advance (§175/2008). Signed informed consent documents were obtained from all of the participants. The research was performed in compliance with the Helsinki Declaration concerning ethical principles. Grants were obtained from both public national funding (VTR-funding) and nonprofit foundations. No conflicts of interest were declared. 

## Results

### Rate of USP non-union

**Figure 2.**
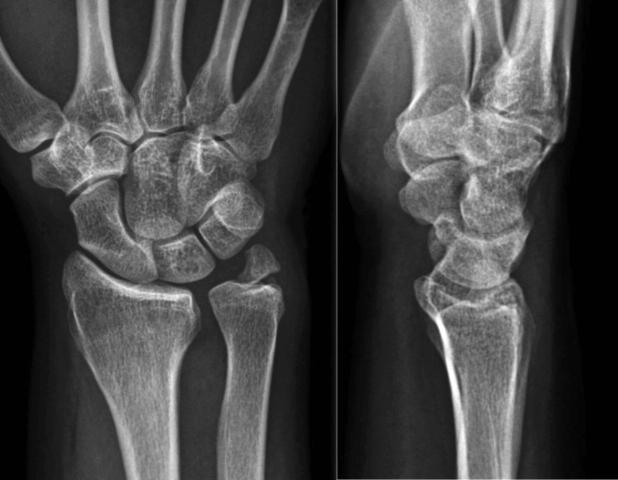
USP non-union 11 years after distal radius fracture.

The rate of USP non-union (Figure 2) following childhood distal forearm fracture was 16% (22/139) at mean 11 years of follow-up. 13 of the 22 fractures that failed to unite had been invisible in the primary radiographs. In the subgroup of 12 cases whose USP fracture was visible in the primary radiographs, 9 did not heal.

### Radiographic findings

7 cases out of the 22 with non-union vs. none in the reference group (N = 117, p < 0.001) showed ulna minus (mean 2.3 mm) when compared with the uninjured side. Respective rate of premature growth plate arrest of the radius was 4% (5/139) and the mean ulna plus was 2.1 mm.

None of the non-union patients showed degenerative radiographic findings. The mean displacement of the non-united fragment was 0.99 mm (SD 0.60, range 0.0–2.3 mm). 8 patients showed fragmented USP in the radiographs.

### Functional findings

There was no decrease in wrist movement in patients with non-union, compared with the respective movement on the uninjured side. Grip strength did not differ between the injured and uninjured wrists (Table 2). 6 patients in the entire cohort showed clinically unstable DRUJ but the rates were similar between the non-union and union groups. 5 patients with USP non-union and 18 in the reference group presented crepitation in the radiocarpal joint (p = 0.4).

**Table 2.** Clinical findings in the injured vs. uninjured extremity in patients with UPS non-union 9–15 years after distal radius fracture (n = 22). Values are mean (SD)

Factor	Injured	Uninjured	p-value^a^
Wrist flexion	80° (5.7)	83° (5.1)	0.2
Wrist extension	80° (10)	80° (10)	1.0
Deviation, ulnar	32° (5.7)	32° (5.7)	1.0
Deviation, radial	20° (5.7)	20° (5.7)	1.0
Grip strength, Nm	47 (16)	49 (15)	0.6

a Independent samples t-test.

### Subjective symptoms

6/22 cases in the non-union group vs. 21/117 in the reference group reported pain during exercise (p = 0.3). No statistically significant difference was found in subjective symptoms between the patients with or without USP non-union at long-term follow-up (Table 3).

**Table 3.** Symptoms found in patients with USP non-union vs. intact USP 11.4 years after distal radius fracture with or without concomitant USP fracture. Values are frequency

Factor	Non-union (n = 22)	Union (n = 117)	p-value^a^
Crepitation	5	18	0.4
Visible deformity	2	0	0.02
Limitation in movement	0	2	1.0
Symptoms in daily life	5	21	0.6
Limb-length discrepancy	0	0	N/A
Subjective dissatisfaction	1	2	0.4
Pain on palpation	1	10	1.0

a Chi-square test and Fisher’s exact test.

### Effect of fracture type, treatment, and immobilization on the risk of ulnar styloid non-union

Displacement of ≥ 2 mm (OR 1.6, CI 0.52–4.9), angular deformity of > 15° (OR 1.2, CI 0.40–4.1), and Salter–Harris classification 2 or more in the radius (OR 2.8, CI 0.4–18) did not increase the risk of non-union. Further, no increased risk was found in connection with longer immobilization time (≥ 28 days) (OR 1.5, CI 0.55–4.1), lower expertise of the physician (OR 1.0, CI 0.37–2.9), or male sex (OR 1.02, CI 0.36–2.9).

Both base (n = 12) and tip (n = 10) fractures were associated with USP non-union but not statistically significantly (p = 0.6). 9 of the 22 USP non-union patients showed ≥ 2 mm anterior-posterior displacement of the radius primarily.

In the subgroup analysis, comparing the proportions of the base and tip factures in patients with primarily diagnosed USP fracture, both types of fracture were equally common among the patients with healed USP fracture and USP non-union.

## Discussion

Ulnar styloid process fracture is associated with 20% of all distal radius fractures in children and it usually fails to unite (Abid et al. 2008). In our population-based study, USP non-union was seen in 16% of all distal radius fractures. Only one-fourth of the primarily recognized USP fractures successfully progressed to ossification during the long 11-year follow-up. Such a low rate of bone union is extremely unusual in childhood fractures. The USP is unique in this regard and the high non-union risk needs to be recognized.

An interesting finding was that a majority of the patients with USP non-union were primarily diagnosed with isolated distal radius fracture but showed USP non-union in the long-term follow-up. In children, the real incidence of USP non-union can only be evaluated after ossification of the ulnar styloid, if MRI is not available. Thus, there may be under-diagnosis of acute USP fractures in young children, which may explain the previously suggested higher incidence of USP non-union in adults (Stansberry et al. 1990, Abid et al. 2008, Wijffels et al. 2014). In our study, the patients were on average 21 years of age (14–29) at the time of follow-up. In that age group, the ulnar styloid process is ossified and visible in radiographs (Gilsanz and Ratib 2005) even though physeal closure usually occurs at the age of 16–19 years (Egol et al. 2010). It is possible that the open physis of the ulna would have made radiographic evaluation more difficult in the youngest study patients. However, reference imaging of the uninjured wrist was undertaken in all cases in order to support radiographic analysis. Routine MR imaging or CT scans were not included in the study plan because they have not been reported to be superior in diagnosing USP fractures, compared with plain radiographs in patients with mature skeleton (Spence et al. 1998, Welling et al. 2008).

We found that the patients with USP fracture showed excellent clinical outcomes after > 10 years of follow-up, regardless of bone healing. Some individual patients reported pain during sports and minor complaints in daily life, but the findings were similar regardless of union or non-union.

Many investigators have suggested an increased risk of DRUJ instability in patients with distal radius fracture with concomitant USP fracture due to lesions in the TFCC (Kazemian et al. 2011, Daneshvar et al. 2014, Gogna et al. 2014). However, we found DRUJ instability in only a few patients and in patients with both normal USP and USP non-union.

Radiographic analysis was performed at a mean of 11 years after the initial injury. The mean residual displacement of the USP was only 1.0 mm. One-third of non-union patients showed ulnar shortening, which may predispose them to later degenerative processes. Ulnar shortening has been associated with ligament disruption (Ramos-Escalona et al. 2010) and early-stage joint degeneration (Kristensen and Soballe 1987, De Smet 1994). Premature radius growth arrest occurred in 4% of our patients, which was expressed as ulnar lengthening, using the uninjured side as a reference. Such a low rate of radius growth plate arrest is in agreement with the previous literature (1% to 7%) (Buterbaugh and Palmer 1988, Cannata et al. 2003, Abzug et al. 2014). It also needs to be kept in mind that radius and ulnar length discrepancy is not a static but a dynamic condition and the relationship changes during forearm rotation and grip loading (Schuurman et al. 2001).

The associated risk factors of USP non-union remain unclear. Previous studies have suggested the base type of USP fracture to be a risk factor (Abid et al. 2008, Zenke et al. 2009), which we did not find. We performed a comprehensive risk analysis on patient-related information including sex, radiographic displacement and angular deformity, severity of the radius fracture according to Salter–Harris classification, treatment method, and the length of immobilization time to evaluate the risk of USP non-union. None of these was associated with a greater risk of USP non-union in this long-term study.

The participation rate of 67% is a weakness of this study. It is probable that some of the non-participants had fewer symptoms than participants and were thus not interested in the follow-up examination. We assume that there were fewer symptomatic patients and non-unions among the non-participants, while a majority of non-participants reported full recovery on their own initiative, when they declined their participation. Nevertheless, it sounds reasonable that the rate of USP non-union could have been smaller with full participation. It is also possible that some of the patients did not participate for reasons unrelated to the analyzed outcome variables in this study (Dunn et al. 2004, Galea and Tracy 2007). Such cases are considered to be missing completely at random, thus having no effect on the results (Kristman et al. 2004). Considering the relatively great time span between the primary injury in childhood and the follow-up visit, the participation rate was satisfactory, according to the literature, and a 60–80% participation rate has been recommended in epidemiologic long-term follow-up studies by many (Kristman et al. 2004, Galea and Tracy 2007, Fewtrell et al. 2008). Further, age, sex, and treatment were similar among participants and non-participants, which strengthens the findings.

As another limitation, the primary data were based on those in hospital registers and not all interesting particulars were available. Further, we found only 22 patients with USP non-union and a larger study would be necessary to confirm possible causal links between the primary injury, its treatment and the risk of USP non-union. In addition, complete DASH data were not utilized, although the baseline scores were collected and analyzed. However, longer follow-up is warranted to make conclusions concerning morbidity in later adulthood.

In summary, although USP non-union was relatively common after a childhood distal radius fracture, the long-term clinical results were good. Ulnar shortening was found in one in three, however, and may predispose an individual to degenerative processes later in life.

LK analyzed the data and wrote the manuscript, SV analyzed all radiographs and critically reviewed the manuscript, WS contributed to the study design and data collection and critically reviewed the manuscript, JJS initiated the study, contributed to study design, carried out the follow-up visits and clinical examinations of the patients, and critically reviewed the manuscript.

The authors would like to thank the Vaasa Foundation of Physicians, Finska Läkaresällskapet, the Emil Aaltonen Foundation, the Finnish Foundation of Pediatric Research and the Alma and K. A. Snellman Foundation for supporting the study. 

## References

[CIT0001] Abid A, Accadbled F, Kany J, de Gauzy J S, Darodes P, Cahuzac J P Ulnar styloid fracture in children: a retrospective study of 46 cases. J Pediatr Orthop B 2008; 17(1): 15–19. 18043372 10.1097/BPB.0b013e3282f3cacb

[CIT0002] Abzug J M, Little K, Kozin S H Physeal arrest of the distal radius. J Am Acad Orthop Surg 2014; 22(6): 381–9. 24860134 10.5435/JAAOS-22-06-381

[CIT0003] Bae D S, Waters P M Pediatric distal radius fractures and triangular fibrocartilage complex injuries. Hand Clin 2006; 22(1): 43–53. 16504777 10.1016/j.hcl.2005.09.002

[CIT0004] Buterbaugh G A, Palmer A K Fractures and dislocations of the distal radioulnar joint. Hand Clin 1988; 4(3): 361–75. 3049631

[CIT0005] Cannata G, De Maio F, Mancini F, Ippolito E Physeal fractures of the distal radius and ulna: long-term prognosis. J Orthop Trauma 2003; 17(3): 180. 10.1097/00005131-200303000-00002 12621255

[CIT0006] Chen Y, Zheng X, Shi H, Wangyang Y, Yuan H, Xie X, Li D, Wang C, Qiu X Will the untreated ulnar styloid fracture influence the outcome of unstable distal radial fracture treated with external fixation when the distal radioulnar joint is stable. BMC Musculoskelet Disord 2013; 14: 186. 23758986 10.1186/1471-2474-14-186 PMC3686660

[CIT0007] Daneshvar P, Chan R, MacDermid J, Grewal R The effects of ulnar styloid fractures on patients sustaining distal radius fractures. J Hand Surg Am 2014; 39(10): 1915–20. 25135248 10.1016/j.jhsa.2014.05.032

[CIT0008] de Putter C E, van Beeck E F, Looman C W N, Toet H, Hovius S E R, Selles R W Trends in wrist fractures in children and adolescents, 1997–2009. J Hand Surg Am 2011; 36(11): 1815.e2. 10.1016/j.jhsa.2011.08.006 22036281

[CIT0009] De Smet L Ulnar variance: facts and fiction review article. Acta Orthop Belg 1994; 60(1): 1–9. 8171975

[CIT0010] Dunn K M, Jordan K, Lacey R J, Shapley M, Jinks C Patterns of consent in epidemiologic research: evidence from over 25,000 responders. Am J Epidemiol 2004; 159(11): 1087–94. 15155293 10.1093/aje/kwh141

[CIT0011] Egol K, Koval K, Zuckerman J Radius and ulna shaft In: Handbook of fractures, 4th ed Philadelphia: Lippincott Williams & Wilkins 2010; p 257–68.

[CIT0012] Fewtrell M S, Kennedy K, Singhal A, Martin R M, Ness A, Hadders-Algra M, Koletzko B, Lucas A How much loss to follow-up is acceptable in long-term randomised trials and prospective studies? Arch Dis Child 2008; 93(6): 458–61. 18495909 10.1136/adc.2007.127316

[CIT0013] Galea S, Tracy M Participation rates in epidemiologic studies. Ann Epidemiol 2007; 17(9): 643–53. 17553702 10.1016/j.annepidem.2007.03.013

[CIT0014] Gilsanz V, Ratib O Hand bone age: a digital atlas of skeletal maturity. Berlin/Heidelberg: Springer Science & Business Media; 2005.

[CIT0015] Gogna P, Selhi H S, Mohindra M, Singla R, Thora A, Yamin M Ulnar styloid fracture in distal radius fractures managed with volar locking plates: to fix or not? J Hand Microsurg 2014; 6(2): 53–8. 25414551 10.1007/s12593-014-0133-7 PMC4235821

[CIT0016] Kazemian G H, Bakhshi H, Lilley M, Emami Tehrani Moghaddam M, Omidian M M, Safdari F, Mohammadpour I DRUJ instability after distal radius fracture: a comparison between cases with and without ulnar styloid fracture. Int J Surg 2011; 9(8): 648–51. 21920472 10.1016/j.ijsu.2011.08.005

[CIT0017] Khosla S, Melton L J, Dekutoski M B, Achenbach S J, Oberg A L, Riggs B L Incidence of childhood distal forearm fractures over 30 years: a population-based study. JAMA 2003; 290(11): 1479–85. 13129988 10.1001/jama.290.11.1479

[CIT0018] Kramer S, Meyer H, O’Loughlin P F, Vaske B, Krettek C, Gaulke R The incidence of ulnocarpal complaints after distal radial fracture in relation to the fracture of the ulnar styloid. J Hand Surg Eur 2013; 38(7): 710–17. 10.1177/1753193412469582 23221179

[CIT0019] Kristensen S S, Soballe K Kienbock’s disease: the influence of arthrosis on ulnar variance measurements. J Hand Surg Br 1987; 12(3): 301–5. 3437194 10.1016/0266-7681_87_90178-1

[CIT0020] Kristman V, Manno M, Cote P Loss to follow-up in cohort studies: how much is too much? Eur J Epidemiol 2004; 19(8): 751–60. 15469032 10.1023/b:ejep.0000036568.02655.f8

[CIT0021] Logan A J, Lindau T R The management of distal ulnar fractures in adults: a review of the literature and recommendations for treatment. Strategies Trauma Limb Reconstr 2008; 3(2): 49–56. 18766429 10.1007/s11751-008-0040-1 PMC2553431

[CIT0022] Mulders M A M, Fuhri Snethlage L J, de Muinck Keizer Robert-Jan O, Goslings J C, Schep N W L Functional outcomes of distal radius fractures with and without ulnar styloid fractures: a meta-analysis. J Hand Surg Eur 2018; 43(2): 150–7. 10.1177/1753193417730323 PMC5791517 28931338

[CIT0023] Nelson O A, Buchanan J R, Harrison C S Distal ulnar growth arrest. J Hand Surg Am 1984; 9(2): 164–70. 6715818 10.1016/s0363-5023(84)80134-3

[CIT0024] Ramos-Escalona J, Garcia-Bordes L, Martinez-Galarza P, Yunta-Gallo A Ulnar variance and scaphoid fracture. J Hand Surg Eur 2010; 35(3): 195–7. 10.1177/1753193409352281 20007423

[CIT0025] Schuurman A H, Maas M, Dijkstra P F, Kauer J M Assessment of ulnar variance: a radiological investigation in a Dutch population. Skeletal Radiol 2001; 30(11): 633–8. 11810155 10.1007/s002560100414

[CIT0026] Souer J S, Ring D, Matschke S, Audige L, Marent-Huber M, Jupiter J B Effect of an unrepaired fracture of the ulnar styloid base on outcome after plate-and-screw fixation of a distal radial fracture. J Bone Joint Surg Am 2009; 91(4): 830–8. 19339567 10.2106/JBJS.H.00345

[CIT0027] Spence L D, Savenor A, Nwachuku I, Tilsley J, Eustace S MRI of fractures of the distal radius: comparison with conventional radiographs. Skeletal Radiol 1998; 27(5): 244–9. 9638833 10.1007/s002560050375

[CIT0028] Stansberry S D, Swischuk L E, Swischuk J L, Midgett T A Significance of ulnar styloid fractures in childhood. Pediatr Emerg Care 1990; 6(2): 99–103. 2371164 10.1097/00006565-199006000-00007

[CIT0029] Waters P M, Bae D S, Montgomery K D Surgical management of posttraumatic distal radial growth arrest in adolescents. J Pediatr Orthop 2002; 22(6): 717–24. 12409894

[CIT0030] Welling R D, Jacobson J A, Jamadar D A, Chong S, Caoili E M, Jebson P J MDCT and radiography of wrist fractures: radiographic sensitivity and fracture patterns. AJR Am J Roentgenol 2008; 190(1): 10–16. 18094287 10.2214/AJR.07.2699

[CIT0031] Wijffels M M E, Keizer J, Buijze G A, Zenke Y, Krijnen P, Schep N W L, Schipper I B Ulnar styloid process nonunion and outcome in patients with a distal radius fracture: a meta-analysis of comparative clinical trials. Injury 2014; 45(12): 1889–95. 25282298 10.1016/j.injury.2014.08.007

[CIT0032] Yuan C, Zhang H, Liu H, Gu J Does concomitant ulnar styloid fracture and distal radius fracture portend poorer outcomes? A meta-analysis of comparative studies. Injury 2017; 48(11): 2575–81. 28882374 10.1016/j.injury.2017.08.061

[CIT0033] Zenke Y, Sakai A, Oshige T, Moritani S, Nakamura T The effect of an associated ulnar styloid fracture on the outcome after fixation of a fracture of the distal radius. J Bone Joint Surg Br 2009; 91(1): 102–7. 19092013 10.1302/0301-620X.91B1.21026

[CIT0034] Zoetsch S, Kraus T, Weinberg A M, Heidari N, Lindtner R A, Singer G Fracture of the ulnar styloid process negatively influences the outcome of paediatric fractures of the distal radius. Acta Orthop Belg 2013; 79 (1): 48–53. 23547515

